# Influencing factors of antibody response after 2 doses of inactivated COVID-19 vaccine among adults aged ≥18 years in Chongqing, China: A cross-sectional serological study

**DOI:** 10.1097/MD.0000000000040075

**Published:** 2024-10-18

**Authors:** Qing Wang, Jiawei Xu, Yu Liu, Jianqiao Li

**Affiliations:** a Expand Program on Immunization, Chongqing Center for Disease Control and Prevention, Chongqing, China.

**Keywords:** COVID-19, inactivated COVID-19 vaccine, influencing factors, neutralizing antibody, pseudovirus-based neutralizing assay

## Abstract

The study aimed to explore the influencing factors after 2 doses of inactivated COVID-19 vaccines (Sinopharm/BBIBP-CorV) in the real world. We conducted a cross-sectional serological study involving 316 volunteers aged ≧ 18 years from 7 vaccination hospitals in the Yubei districts, Yuzhong districts, and Jiulongpo districts of Chongqing. Serum samples were obtained about 1 month after 2 dose vaccination, and Nabs were tested using the pseudovirus-based neutralizing assay. Chi-square or Fisher exact tests were used to analyze the seropositive rates, while the Kruskal–Wallis *H* or Mann–Whitney *U* tests were used to analyze differences in Nabs level among stratified groups. Logistic regression analyses were conducted to identify the influencing factors. The results showed that seropositive rates was 76.27% and the GMT was 26.13 (95% CI: 23.03–29.66) after 2 doses of COVID-19 inactivated vaccination. The risk of being seropositive in 18 to 29, 30 to 39, 40 to 49, 50 to 59, and 60 to 69 years were 12.808-fold, 8.041-fold, 7.818-fold, 6.275-fold, 1.429-fold compared with the people aged ≥ 70 years (*P* < .05), and the risk of being seropositive of intervals 15 to 21 and 22 to 28 days were 0.273-fold and 0.286-fold compared with >28 days (*P* < .05), respectively. In conclusion, age may be a risk factor for reduced antibody production, and longer vaccination intervals-may be a protective factor that increases antibody production. These findings contribute to informing future vaccination strategies.

## 1. Introduction

Coronavirus disease 2019 (COVID-19) is an infectious disease caused by severe acute respiratory syndrome coronavirus 2 virus (SARS-CoV-2), had caused significant damage to global health and the economy.^[1]^ To prevent the pandemic of COVID-19, multiple technical platforms for COVID-19 vaccines (inactivated vaccines, recombinant protein vaccines, adenovirus-vector vaccines, and mRNA vaccines) have been developed in China. The clinical study showed that COVID-19 inactivated vaccine is safe and has induced antibody response in adults.^[2–4]^ Vaccine-induced neutralizing antibodies (Nabs) are key biomarkers considered to be associated with vaccine efficacy, but the level of Nabs produced can vary significantly among individuals, influenced by diverse factors such as age, gender, and comorbidities.^[5]^ However, the data on factors influencing the Nabs production of inactivated vaccines in the real world are scarce. Hence, we conducted a serological investigation to explore the factors influencing Nabs production in individuals aged ≥ 18 years after 2 doses Sinopharm/BBIBP-CorV vaccine to provide reference for optimizing vaccination regimen.

## 2. Materials and methods

### 2.1. Participants

According to a previous clinical study, the seropositive conversion rate of people aged ≧ 18 years after vaccination is between 92% and 100%.^[2]^ The seropositive rate (*P*) is estimated to be 96% for sample size calculation in this study, with a confidence level (1-α) reflecting the degree of confidence at 0.95 and a maximum allowable error not exceeding (d) 6%, the minimum effective sample size is calculated by PASS15.0 to be 275 individuals. Considering a 15% loss to follow-up and invalid samples, the total sample size was calculated to be 316 study participants.

The inclusion criteria were no contraindications to vaccination with age ≥ 18 years primed with 2 doses of BBIBP-CorV vaccine. Those were excluded: previously infected with SARS-CoV-2; with COVID-19 symptoms such as fever, cough, fatigue, etc; pregnant women; co-infected with HIV/HCV; immunocompromised.

### 2.2. Method

Between February 9, 2021 and December 3, 2021, we conducted a cross-sectional serological study. We recruited healthy volunteers from 7 vaccination hospitals in the Yubei District, Yuzhong District, and Jiulongpo District of Chongqing. We collected serum samples 1 month (26–59 days) after vaccination with 2 doses of COVID-19 inactivated vaccine. Meanwhile, we designed a questionnaire included basic information and health information (age, sex, comorbidity) of the volunteer, vaccination information (vaccination type and time), and blood collection time, which was obtained by the vaccination doctor's query. Vaccination information was obtained from the Chongqing Immunization Planning Information Management System. Comorbidity for those aged 60 and over was obtained from primary care services in China.

### 2.3. Vaccinations

The primary vaccination schedule of BBIBP-CorV vaccine is 2 doses, each dose interval 14 to 28 days, with 0.5 mL of each dose injected intramuscularly into the deltoid muscle of the upper arm.

### 2.4. Pseudovirus-based neutralization assay (PBNA)

The laboratory testing was conducted in the Biosafety Level 2 Laboratory. All serum samples were tested for neutralizing antibodies against the Wuhan-1 strain. The PBNA for SARS-Cov-2 was established by the National Institutes for Food and Drug Control of China,^[6,7]^ and the SARS-CoV-2 pseudovirus was prepared and provided by Guangzhou Darui Biotechnology Co., Ltd (Darui Biotech, Guangzhou, China). The 50% tissue culture infectious dose (TCID_50_) was determined by the transduction of pseudovirus into Huh7 cells (JCRB). For the pseudovirus neutralization assay, 100 µL of human serum samples at different concentrations (1:10 to 1:2430) were mixed with 50 µL supernatant containing 650 to 1300 TCID_50_ SARS-CoV-2 pseudovirus. The mixture was incubated for 1 h at 37°C, supplied with 5% CO_2_ After the incubation, 100 µL of Huh7 cell suspension (3 × 10^5^ cells/mL) were then added to the mixtures of pseudoviruses and diluted serum for an additional 20 to 28 h incubation in the 37°C, 5% (vol/vol) CO_2_ incubator. Then, 150 µL of supernatant was removed, and 100 µL of luciferase detection reagent (Darui Biotech) was added to each well. After 2 minutes of incubation, each well was mixed 10 times by pi-petting, and 150 µL of the mixture was transferred to a new white micro-plate. Luciferase activity was measured using a microplate luminometer (PerkinElmer). Then, the infection inhibition rates of each dilution of the sample were calculated according to the relative luminescence unit (RLU) values as follows: inhibition rate = [1–(average RLU of sample–average RLU of CC)/(average RLU of VC–average RLU of CC)]×100% (CC = cell control, VC = virus control). Based on the inhibition rate results, the 50% inhibitory dilution (EC_50_) of each sample was calculated by the Reed–Muench method. The values of EC_50_ titer ≥ 10 were considered positive.

### 2.5. Statistical analysis

Statistical analysis was performed SPSS 21.0. The seropositivity rates and geometric mean titers (GMT) of Nabs were the main indicators in this study. The dependent variable was seropositivity and 6 independent variables included sex, age, diabetes, hypertension, vaccination days and blood collection days in our study, with continuous variables classified as categorical variables. Age was divided into 6 groups for each additional 10 years. Vaccination days were divided into 3 groups for each additional 7 days, and blood collection days were divided into 2 groups according to the blood collection days of 50% of the subjects. The categorical data were described with the frequency and percentage. Seropositivity rates were compared using the Chi-square test or Fisher exact test. Log10-converted EC_50_ values were compared using the Mann–Whitney *U* test or Kruskal–Wallis *H* test, binomial logistic regression was used to analyze the effect of factors. The EC_50_ ≥ 10 was defined as positive, EC50 < 10 was defined as negative with valve as 5, and 2-sided *P* values <0.05 were considered statistically significant.

### 2.6. Ethical consideration

This study was approved by the Ethics Committee of the Chongqing Center for Disease Control and Prevention (No. KY-2022-015-2). All participants were informed of the purpose of the study and written informed consent before enrollment.

## 3. Results

### 3.1. Basic information and health information of participants

A total of 316 volunteers participated in this serologic survey, ranging from 19 to 82 years with a mean age of 48.22 ± 17.23 years. Among them, 94 (29.75%) were males and 222 (70.25%) were females. The age distribution was as follows: 50 (15.82%) were aged between 18 and 29 years, 73 (23.10%) were aged between 30 and 39 years, 54 (17.09%) were aged between 40 and 49 years, 39 (12.34%) were aged between 50 and 59 years, 49 (15.51%) were aged between 60 and 69 years, and 51 (16.14%) were aged ≧ 70 years. Among the participants, 40 (12.66%) had hypertension, 24 (7.59%) had diabetes, 38 (12.03%) completed vaccinations between 15 and 21 days, 120 (37.97%) completed vaccinations between 22 and 28 days, and 158 (50.00%) completed vaccinations > 28 days. For serum samples, 195 (61.71%) were obtained between 26 and 31 days, and 121 (38.29%) were obtained between 32 and 44 days. There were no missing data, and all volunteers were included in the analysis.

### 3.2. Differential analysis of seropositive rates and GMT

Our study observed a seropositive rates of 76.27% and a GMT of neutralizing antibodies of 26.13 (95% CI: 23.03–29.66) after 2 doses of the COVID-19 inactivate vaccination. The seropositive rates of females were 81.53% and GMT 29.53 (95% CI: 25.53–34.14), and those of males were 63.83% and the GMT 19.60 (95% CI: 15.33–25.06). The females had a higher seropositive rates and GMT compared to the males (*P* = .001, *P* = .007). The seropositive rates were 94.00%, 86.30%, 88.89%, 89.74%, 51.02%, and 45.10%, and the GMT were 47.74 (95% CI: 36.02–63.27), 31.11 (95% CI: 24.36–39.73), 35.28 (95% CI: 27.13–45.89), 40.29 (95% CI: 28.84–56.30), 13.25 (95% CI: 9.69–18.12), and 11.33 (95% CI: 8.64–14.87) in the 18 to 29 years, 30 to 39 years, 40 to 49 years, 50 to 59 years, 60 to 69 years, and ≥70 years groups, respectively. A significant decrease in seropositivity rates and GMT was observed with increasing age, especially in those aged 60 years and older (*P* < .001, *P* < .001). Regarding comorbidities, individuals with diabetes or hypertension demonstrated lower seropositive rates (41.67% for diabetes, 50.00% for hypertension) and GMT (10.18 [95% CI: 6.95–14.89 for diabetes], 13.66 [95% CI: 9.77–19.12 for hypertension]) compared to those without these conditions (79.11% and GMT 28.24 (95% CI: 24.80–32.16) for those without diabetes, 80.74% and GMT 29.19 (95% CI: 25.56–33.34) for those without hypertension) (*P* < .001, *P* < .001). Furthermore, the seropositive rates for vaccination intervals of 15 to 21, 22 to 28, and >28 days were 55.26%, 67.50%, and 87.34%, and with GMT 13.25 (95% CI: 9.55–18.40), GMT 19.30 (95% CI: 15.80–23.57), and GMT 38.74 (95% CI: 32.81–45.75), respectively. The longer vaccination intervals were associated with an increase in seropositive rates and GMT (*P* < .001, *P* < .001). The seropositive rates for blood collection intervals of 26 to 31 days and 32 to 44 days were 64.29% and 53.70%, with GMT of 18.32 (95% CI: 15.00–22.38) and 15.03 (95% CI: 12.42–18.20), respectively, but no statistically significant differences were found (Table 1).

**Table 1:** Differential analysis of seropositive rates and GMT in different characteristic population (N stands for the number of samples,% represents the seropositive rates of serum).

Characteristics	N	EC_50_ ≥ 10 (%)	*X^2^*	*P*	GMT	95% CI, low to up	*Z/H*	*P*
Sex								
Male	94	60 (63.83)	11.432	.001	19.60	15.33 to 25.06	−2.705	.007
Female	222	181 (81.53)			29.53	25.53 to 34.14		
Age (yr)								
18 to 29	50	47 (94.00)	66.039	<.001	47.74	36.02 to 63.27	66.413	<.001
30 to 39	73	63 (86.30)			31.11	24.36 to 39.73		
40 to 49	54	48 (88.89)			35.28	27.13 to 45.89		
50 to 59	39	35 (89.74)			40.29	28.84 to 56.30		
60 to 69	49	25 (51.02)			13.25	9.69 to 18.12		
≥70	51	23 (45.10)			11.33	8.64 to 14.87		
Diabetes								
No	292	231 (79.11)	17.777	<.001	28.24	24.80 to 32.16	−4.107	<.001
Yes	24	10 (41.67)			10.18	6.95 to 14.89		
Hypertension								
No	270	218 (80.74)	20.519	<.001	29.19	25.56 to 33.34	−4.073	<.001
Yes	46	23 (50.00)			13.66	9.77 to 19.12		
Vaccination interval (d)								
15 to 21	38	21 (55.26)	26.321	<.001	13.25	9.55 to 18.40	38.721	<.001
22 to 28	120	81 (67.50)			19.30	15.80 to 23.57		
˃28	158	139 (87.34)			38.74	32.81 to 45.75		
Blood collection interval (d)								
26 to 31	140	90 (64.29)	3.467	.063	18.32	15.00 to 22.38	−1.591	.112
32 to 44	162	87 (53.70)			15.03	12.42 to 18.20		
Total	316	241 (76.27)			26.13	23.03 to 29.66		

EC_50_** = **50% inhibitory dilution.

### 3.3. Logistic regression analysis of influencing factors

We performed a univariate and multivariate binary logistic regression analysis, considering Nabs positivity as the dependent variable and sex, age, diabetes, hypertension, vaccination interval, and blood collection interval as independent variables (Table 2). Our findings revealed that age was a significant inhibitor of neutralizing antibody production, whereas longer vaccination intervals were protective factors of neutralizing antibody production (*P* < .001, *P* = .001). The risk of being seropositive in 18 to 29, 30 to 39, 40 to 49, 50 to 59, and 60 to 69 years were 12.808-fold, 8.041-fold, 7.818-fold, 6.275-fold, 1.429-fold compared with the people aged ≥ 70 years, and the risk of being seropositive of intervals 15 to 21 and 22 to 28 days were 0.273-fold and 0.286-fold compared with >28 days (*P* < .05), respectively (Table 3).

**Table 2:** Assignment of variables in logistic regression analysis.

Factors	Variable	Value
Neutralizing antibody positivity	Y	Negative = 0, positive = 1
Gender	X1	Male = 1, female = 2
Age (yr)	X2	18 to 29 yr = 1, 30 to 39 yr = 2, 40 to 49 yr = 3, 50 to 59 yr = 4, 60 to 69 yr = 5, ≥70 yr = 6
Diabetes	X3	No = 0, yes = 1
Hypertension	X4	No = 0, yes = 1
Vaccination intervals (d)	X5	15 to 21 d = 1, 22 to 28 d = 2, ˃28 d = 3
Blood collection interval (d)	X6	26 to 31 d = 1, 32 to 44 d = 2

Categorical boundaries for continuous variables.

Age: there are 6 age groups in total, which are divided into 1 age group for each additional 10 yr – 18 to 29, 30 to 39, 40 to 49, 50 to 59, 60 to 69, and ≥70 yr.

Vaccination interval: each additional 7 d was divided into vaccination interval, and a total of 3 vaccination intervals were divided into 3 vaccination intervals – 15 to 21, 22 to 28, and >28 d.

Blood collection interval: according to the blood collection days of 50% volunteers, it was divided into 2 sections – 26 to 31 and 32 to 44 d.

**Table 3:** Univariate and multivariate logistic regression analysis of factors influencing on neutralizing antibodies after 2 doses of the vaccinations (N = 316).

Characteristics	Univariate		Multivariate				
	Exp (B)	*P*	B	*P*	Exp (B)	95% CI-low	95% CI-up
Sex	2.502	.001	−0.467	.161	0.627	0.326	1.205
Age (yr)		<.001		<.001			
18 to 29 versus ≥70 (references)	19.072	<.001	2.550	<.001	12.808	3.181	51.566
30 to 39 versus ≥70 (references)	7.670	<.001	2.085	<.001	8.041	2.921	22.136
40 to 49 versus ≥70 (references)	9.739	<.001	2.056	<.001	7.818	2.488	24.567
50 to 59 versus ≥70 (references)	10.652	<.001	1.837	.006	6.275	1.703	23.121
60 to 69 versus ≥70 (references)	1.268	.554	0.357	.416	1.429	0.605	3.375
Diabetes	0.189	<.001	0.385	.478	1.470	0.507	4.259
Hypertension	0.239	<.001	−0.130	.769	0.879	0.371	2.082
Vaccination interval (d)		<.001		.001			
15 to 21 versus ˃28 (references)	0.169	<.001	−1.298	.007	0.273	0.107	0.698
22 to 28 versus ˃28 (references)	0.284	<.001	−1.251	.001	0.286	0.139	0.591
Blood collection interval	0.511	.012	−0.082	.805	0.922	0.482	1.763
Constant			0.612	.300	1.844		

## 4. Discussion

Currently, virus neutralization assays are critical for the development and evaluation of vaccines. However, neutralization assays traditionally require the use of infectious virus which must be carefully handled in BSL-3 setting, thus complicating the assay and restricting its use.^[8]^ Conversely, Based on a VSV pseudovirus production system, a PBNA has been developed for evaluating Nabs against SARS-CoV-2 in BSL-2 laboratories, broadening their accessibility and emerging as a widely adopted testing strategy for the clinical assessment of vaccines.^[9,10]^

The findings of the study demonstrate that as age increases, there is a corresponding decrease in both the seropositive rates and GMT of Nabs. Notably, a significant reduction of Nabs was observed in individuals aged 60 years and older. Furthermore, the pattern of age-related changes observed in this study aligns with the results of previous study.^[11–13]^ Immune senescence may reduce vaccine response in the elderly, possibly due to the changes in the immune organs most notably in the thymus, with reduced activity of thymocytes and thymic epithelial cells, and a decrease in the immune response substances, which leads to a decrease in immune function.^[14]^ In addition, the production of activated B cells and the function of immunoglobulins are important issues.^[15]^

Previous research has suggested that diabetes can lead to a dysfunctional immune response, which may compromise the ability to control the spread of invading pathogens in diabetic patients.^[16]^ Separately, a study^[17]^ has demonstrated a significant association between hypertension and lower antibody titer. However, we have not observed statistically significant differences in antibody levels among individuals with diabetes or hypertension. This may be due to the fact that the number of volunteers with comorbid diabetes and hypertension was too small to observe significant differences.

Our research showed that vaccination interval more than 28 days produced a higher Nabs titers. Previous clinical studies showed that^[4]^ the level of Nabs induced by the vaccination schedule of 0, 21 days and 0, 28 days was higher than that of 0, 14 days. Furthermore, research on mRNA vaccines^[18]^ highlighted that an interval exceeding 25 days between doses considerably augmented the antibody response elicited by the vaccine. It may be related to the longer vaccination interval and the longer antibody maturation time of the receptor.^[19]^

Our study has several limitations. First, we did not perform prevaccination Nabs testing because the COVID-19 pandemic was well controlled, and few were infected in Chongqing in 2021. We assumed the volunteers were healthy and no SARS-CoV-2 infected history, so we could not find the asymptomatically infected people who may have affected the test results. Second, according to PBNA, the lower limit of detection was 30, and we set the lower limit of detection for the test at 10, mainly because we thought that a detection threshold of 10 may be more appropriate based on the characteristics of the inactivated vaccine response. Third, our study did not collect data on the lifestyle and BMI of the volunteers, and we did not perform internal and external validation of the regression models. Given these limitations, we couldn’t find more confounding factors, evidence from multicenter studies is still needed to develop further vaccination strategies.

## 5. Conclusions

Our preliminary findings of age as a risk factor for Nabs production and extended vaccination intervals as a protective factor for Nabs production and provide data to the use of inactivated COVID-19 vaccine in the real world. The World Health Organization recommends that older adults, comorbidity, and immunocompromised individuals who have received 2 doses of the inactivated vaccine should receive an additional dose to ensure adequate protection. The continued development of vaccines containing variant strains and the administration of booster vaccinations are crucial in reducing the risk of severe illness and death from infection in this population. In the future, we will continuously dynamically monitor the influencing factors of antibody response after booster vaccination and conduct cohort studies with larger sample sizes to validate these influencing factors.

## Acknowledgments

We are grateful to the CDCs and vaccinators who provided the help of questionnaire survey and serum sample collection throughout the project, and to Guangzhou Dari Biotechnology Co. for conducting the neutralizing antibody test in serum samples.

## Author contributions

**Conceptualization:** Qing Wang, Jianqiao Li, Jiawei Xu.

**Funding acquisition:** Qing Wang.

**Project administration:** Qing Wang, Jianqiao Li, Jiawei Xu.

**Visualization:** Jianqiao Li, Yu Liu.

**Writing – original draft:** Jianqiao Li, Yu Liu.

**Writing – review & editing:** Qing Wang, Jianqiao Li, Jiawei Xu, Yu Liu.
